# An Autophagy Modulator Peptide Prevents Lung Function Decrease and Corrects Established Inflammation in Murine Models of Airway Allergy

**DOI:** 10.3390/cells10092468

**Published:** 2021-09-18

**Authors:** François Daubeuf, Nicolas Schall, Nathalie Petit-Demoulière, Nelly Frossard, Sylviane Muller

**Affiliations:** 1 CNRS-Strasbourg University Laboratoire d’Innovation Thérapeutique/Strasbourg Drug Discovery and Development Institute (IMS), Faculté de Pharmacie, 67400 Illkirch, France; francois.daubeuf@unistra.fr (F.D.); nathalie.petit-demouliere@unistra.fr (N.P.-D.); nelly.frossard@unistra.fr (N.F.); 2 CNRS UMS3286, Plate-Forme de Chimie Biologique Intégrative de Strasbourg/Strasbourg Drug Discovery and Development Institute (IMS), 67400 Illkirch, France; 3 CNRS-Strasbourg University Unit Biotechnology and Cell Signaling/Strasbourg Drug Discovery and Development Institute (IMS), Ecole Supérieure de Biotechnologie de Strasbourg, 67400 Illkirch, France; n.schall@unistra.fr; 4 Fédération Hospitalo-Universitaire OMICARE, Fédération de Médecine Translationnelle de Strasbourg, Strasbourg University, 67000 Strasbourg, France; 5 University of Strasbourg Institute for Advanced Study, 67000 Strasbourg, France

**Keywords:** allergic asthma, murine models, autophagy, neutrophils, peptide-based treatment

## Abstract

The involvement of autophagy and its dysfunction in asthma is still poorly documented. By using a murine model of chronic house dust mite (HDM)-induced airway inflammation, we tested the expression of several autophagy markers in the lung and spleen of asthma-like animals. Compared to control mice, in HDM-sensitized and challenged mice, the expression of sequestosome-1/p62, a multifunctional adaptor protein that plays an important role in the autophagy machinery, was raised in the splenocytes. In contrast, its expression was decreased in the neutrophils recovered from the bronchoalveolar fluid, indicating that autophagy was independently regulated in these two compartments. In a strategy of drug repositioning, we treated allergen-sensitized mice with the therapeutic peptide P140 known to target chaperone-mediated autophagy. A single intravenous administration of P140 in these mice resulted in a significant reduction in airway resistance and elastance, and a reduction in the number of neutrophils and eosinophils present in the bronchoalveolar fluid. It corrected the autophagic alteration without showing any suppressive effect in the production of IgG1 and IgE. Collectively, these findings show that autophagy processes are altered in allergic airway inflammation. This cellular pathway may represent a potential therapeutic target for treating selected patients with asthma.

## 1. Introduction

Dust mite represents a major source of allergen in house dust and appears as a key trigger of allergic respiratory disease [1,2], with *Dermatophagoides pteronyssinus* as the main species [2]. Der p 1, a cysteine protease, and Der p 2, a lipopolysaccharide (LPS)-binding protein, are considered as the most potent house dust mite (HDM) allergens. They are abundant in the bodies and fæces of mites, and immunoglobulin (Ig)E reactivity can reach up to 80–90% in HDM-allergic patients. The major allergenic effects in HDM allergy result from both adaptive immune mechanisms through CD4^+^ Th2 cells that induce and drive IgE-dependent allergic responses, and innate immune reactions. Th2 cells represent polarised, differentiated T helper cells, which in addition to interleukin (IL)5, produce IL-4 and IL-13 that are responsible in B cells for the antibody class switch from IgM or IgG antibodies to IgE [3]. Current therapeutic options involve allergen avoidance, pharmacological intervention (e.g., anti-histamine, corticosteroids, therapeutic antibodies to IgE, IL-4/IL-13 receptor, IL-5, IL-5R, thymic stromal lymphopoietin) and allergen immunotherapy [3,4,5,6,7,8,9]. β2-adrenergic receptor agonists that cause smooth muscle dilation (e.g., salbutamol) are mostly used to relieve the symptoms of asthma. In general, these treatments are given all along life of affected patients, leading to unwanted secondary effects in certain individuals and unresponsiveness effects at long term.

Autophagy is an evolutionarily conserved, finely regulated, catabolic process that is vital in the maintenance of normal cell homeostasis [10,11,12,13,14,15]. It exerts a primary role in coordinating cell metabolism and growth with environmentally induced stress. It thus plays an eminent role in the cell physiology. Hence, autophagy participates in the immunopathology of inflammatory diseases. Some alterations—either upregulation or downregulation—in several autophagy pathways, have thus been implicated in numerous (auto)immune and inflammatory disorders, such as systemic lupus erythematosus, Sjögren’s syndrome (SS), rheumatoid arthritis, psoriasis, some neuroinflammatory and neurodegenerative diseases including multiple sclerosis, chronic inflammatory demyelinating polyneuropathies, amyotrophic lateral sclerosis, and Huntington’s, Alzheimer’s, and Parkinson’s diseases [9,16,17,18,19,20,21,22,23,24,25] and also in metabolic diseases, cancer, infection and ageing. In asthma, results are still scarce and interrelationships between asthma and autophagy remain poorly established [26,27,28]. Genetic association studies in independent cohorts of patients have suggested a link between asthma pathogenesis and autophagy related (ATG) to gene 5 [27]. Recent data argue that autophagy is abnormally activated with an increased expression of BECLIN-1 and ATG5, and a reduced expression of sequestosome-1 (SQSTM1)/p62, in lung tissues from certain asthmatic patients lead to airway remodeling effects [28]. However, the role of ATG5, which is an essential element in the initiation steps of the autophagy process, remains controversial due to its implication in several pathways. Autophagy activity also plays a central role in neutrophil and eosinophil functions, as well as in the neutrophil extracellular traps (NETs) and eosinophilic extracellular traps release, which is involved in asthma severity by damaging airway epithelium and inducing inflammatory responses [26,29,30,31].

In the present study conducted in two independent mouse models of allergen-induced airway inflammation, we explored the therapeutic potential of a peptide called P140, known in vitro and in vivo to selectively target chaperone-mediated autophagy (CMA) and to alter the macroautophagy process [32,33,34]. This synthetic phosphopeptide, issued from the cognate sequence 131–151 of the U1-70K spliceosomal protein, encompasses a phosphoserine residue at position 140. It interacts with the ATPase domain (N-terminal nucleotide-binding domain) of the chaperone protein HSPA8, leading in vitro to alterations of the HSPA8-HSP90AA1-bearing heterocomplex integrity, a loss of HSPA8 folding functions by a mechanism that likely involves HSPA8 ATPase activity, and in cellulo affects the vital nuclear translocation of HSPA8 and associated client proteins in heat-shocked cells [32,34]. Using highly purified lysosomes extracted from the liver of MRL/lpr mice that received the P140 peptide intravenously (i.v.), it was shown that P140 regulates CMA at the step of substrate lysosomal uptake [33]. P140 has been evaluated in advanced clinical trials for treating patients with systemic lupus erythematosus and showed a remarkable safety profile [35]. From a mechanistic point of view, it is established in mice and humans that P140 downregulates hyperactive autophagy processes and major histocompatibility complex (MHC)-II molecules expression, which is raised in lupus settings with downstream effects on T- and B-cell activation [32,33,36,37]. This peptide was also shown to prevent NET release in vitro [38]. The beneficial effect of P140 in other chronic inflammatory conditions in which the same processes of autophagy are abnormally activated has also been demonstrated in murine models of primary and secondary SS [36,39] and chronic inflammatory demyelinating polyneuropathy (CIDP) [40]. Asthma is the first non-autoimmune inflammatory indication that is investigated with this therapeutic peptide. Using both a chronic model of HDM-induced asthma features [41] and a model of acute eosinophilic airway inflammation induced by ovalbumin (OVA) [42], we show here that P140 is clinically effective in these experimental settings in terms of a reduction in airway inflammation, as well as airway resistance and elastance in the chronic model. We demonstrate further that autophagy processes are altered in the lungs and spleen of HDM-sensitized mice that develop chronic allergic airway features. Mechanistically, we point out that P140 corrects the abnormal expression of SQSTM1 both in the neutrophils recovered from the bronchoalveolar lavage fluid (BALF) and in the splenocytes of HDM-challenged animals. These results strongly support the view that the autophagy modulator peptide P140 might help decreasing allergic inflammation, and warrants further study in selected patient populations with chronic airway allergic inflammatory disease.

## 2. Materials and Methods

### 2.1. Peptides

The P140 (RIHMVYSKRpSGKPRGYAFIEY) and the scrambled (Sc) P140 (YVSRYFGpSAIRHEPKMKIYRG) phosphopeptides (pS standing for phosphoserine residues) were synthesized as described previously [43]. The homogeneity of peptides was checked by analytical high-performance liquid chromatography and their identity was assessed by mass spectrometry.

### 2.2. Mouse Models of Allergic Asthma

**Figure 1:**
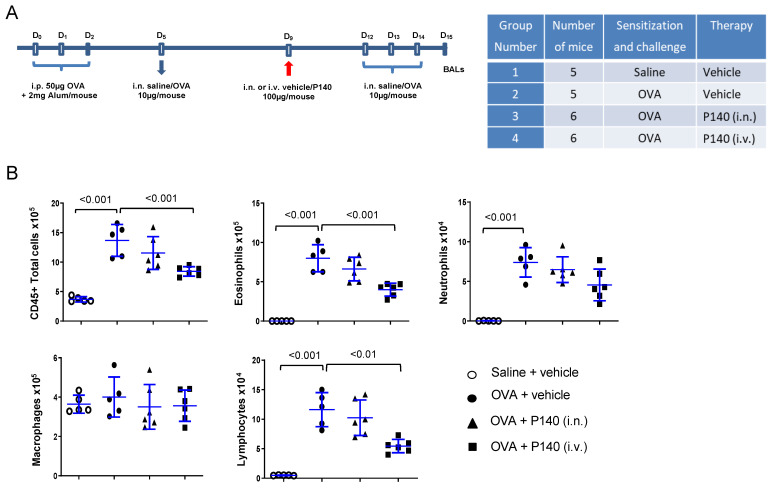
Effect of the P140 peptide on airway inflammatory cell accumulation in an ovalbumin-induced airway hypereosinophilia model in BALB/c mice. (**A**) 9-week old male BALB/c mice were sensitized to OVA in saline at days 0, 1 and 2 and then successively challenged with OVA at days 5, 12, 13 and 14. P140 in vehicle (NaCl) was administered i.n. or i.v. at the dose of 4 mg/kg on day 9. BALs were collected at day 15. (**B**) Absolute numbers of total cells, eosinophils, neutrophils, macrophages and lymphocytes in BALs are shown. The data are presented as the mean ± SEM (*n* = 5–6 mice per group).

**Figure 2:**
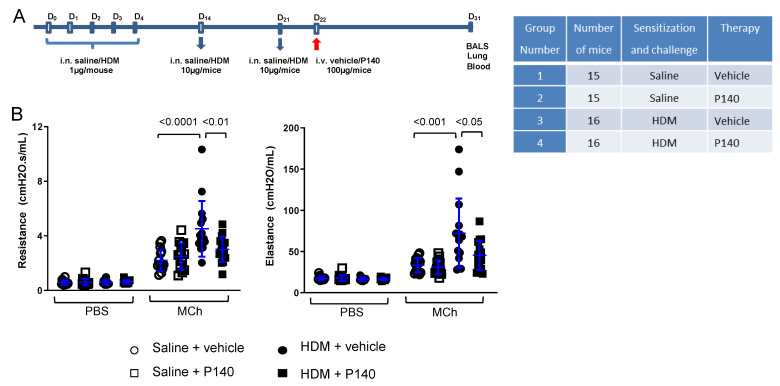
The effect of the P140 peptide in a chronic model of HDM-induced airway inflammation in BALB/c mice. (**A**) 9-week-old female BALB/c mice were sensitized on days 0, 1, 2, 3, 4 by i.n. administration of HDM extract (Stallergenes; 1 µg extract in 25 µL saline). They were challenged with 10 µg extracts on days 14 and 21. Mice were treated by i.v. injection (4 mg/kg body weight) of P140 or vehicle alone on day 22. BALs, lung tissues (total or washed), and blood were collected at day 31. (**B**) Airway resistance (R, cmH_2_O.s.mL^−1^) and airway elastance (cmH_2_O.mL^−1^) in response to aerosolized PBS and MCh (50 mg/mL, i.e., 0.26 M, in PBS) assessed by FlexiVent technique in anaesthetized mice. The data are presented as the mean ± SEM (*n* = 15–16 mice per group from two independent experiments).

BALB/c mice were provided by Janvier Labs (Le Genest St Isle, France). Experiments with OVA and HDM models were conducted as described previously [41,44,45]. Briefly, in the OVA model, mice were sensitized by i.p. administration of 50 µg OVA (Sigma-Aldrich, Saint Quentin Fallavier, France; A5503) mixed with 2 mg alum (Sigma-Aldrich, 23918-6) in saline. Then, mice were challenged by intranasal (i.n.) instillations of 10 µg OVA in saline or saline alone for controls (Figure 1A). For the HDM model, mice were sensitized and challenged by i.n. instillations with 1 µg or 10 µg HDM (Stallergenes-Greer, Antony, France, Der p1) in saline or saline alone for controls (Figure 2A). The P140 or ScP140 peptides were administered once by the i.n. (1 mL/kg) or i.v. (4 mL/kg) route at the dose of 4 mg/kg body weight. At the end of the experiments, airway responsiveness, i.e., airway resistance and elastance, was measured, and BALF, blood and lung tissue were collected [46]. Several independent experiments were carried out over four years strictly using the same HDM-based protocol, allowing us therefore to pool the data and to exclude any possible external (e.g., seasonal, environmental, microbiota) influence on the final results. Animals were maintained under controlled environmental conditions (20 ± 2 °C) in conventional husbandry and a 12 h/12 h light-dark cycle (lighting 7:00 a.m.–7:00 p.m.) was imposed. Mice were kept in large polycarbonate cages with 8 to 10 mice per cage (PCT3, Allentown) with bedding made from spruce wood chips (Safe) and enriched with play tunnels which were changed every week.

### 2.3. Airway Response to Methacholine (Flexivent)

Airway responses to phosphate-buffered saline (PBS) and methacholine (MCh) were assessed using the forced oscillation technique (FlexiVent, SCIREQ, Montreal, Canada) as described [47]. Mice were anesthetized with an intraperitoneal (i.p.) injection of xylasine (Rompun; 1 mg/kg), followed 15 min later by an i.p. injection of pentobarbital sodium (3.64 mg/kg). The trachea was exposed and an 18-gauge metal needle was inserted into the trachea. Airways were connected to a computer-controlled small animal ventilator, and quasi-sinusoidally ventilated with a tidal volume of 10 mL/kg at a frequency of 150 breaths/min and a positive end expiratory pressure of 2 cm H_2_O to achieve a mean respiratory volume close to that of spontaneous breathing. After baseline measurement, each mouse was challenged for 10 s with an aerosol of PBS generated with an in-line nebulizer and administered directly through the ventilator. Then, aerosolized MCh at 50 mg/mL was administered for 10 s. The effect of MCh was calculated as the peak response, i.e., the mean of the three maximal values integrated for the calculation of airway resistance (R, cm H_2_O.s.mL^−1^) and elastance (E, cm H_2_O.mL^−1^).

### 2.4. Flow Cytometry

Differential cell counts were assessed by flow cytometry (BD LSRFortessa™ X-20 Cell Analyzer, BD Biosciences). BAL cells were added with Fixable Viability Stain 450 (562247, BD Biosciences) in a 96 V black microplate, incubated for 20 min at room temperature protected from light. After washing, Fc Block (553142, BD Biosciences) was added for another 20 min incubation time and after a new washing, membrane marker antibodies were finally added: CD11b-APC-Cy7 (557657), CD11c-PE-Cy7 (558079), F4/80-BV510 (743280) from BD Biosciences, Gr-1-PE-eFluor 610 (61-5931-82, eBioscience, Illkirch, France), CD45-Alexa (AF)700 (103128, BioLegend, Paris, France). Antibodies were incubated with BAL cells for 30 min at room temperature. After washing, microtubule-associated proteins 1A/1B light chain 3B (MAP1LC3B) and SQSTM1 expression were measured using the Autophagy detection reagent pack from Millipore (CF200097). Briefly, “reagent B” from Millipore was added to each well and cells were directly centrifuged 5 min at 300× *g*. Cells were washed with “assay buffer”, MAP1LC3B-FITC (from kit) and SQSTM1-AF647 (ab194721, Abcam, Cambridge, UK) were added to each well for 30 min protected from light. After a last wash, cells are resuspended in an “assay buffer” and flow cytometry was performed immediately.

### 2.5. Enzyme-Linked Immunosorbent Assay (ELISA)

Cytokine/chemokine semi-quantification was performed at the PCBIS-TechMed’ILL platform (Illkirch, France) by ELISA in lung homogenates according to manufacturer’s instruction (BD Opteia and R&D Quantikine). For measuring HDM-specific IgE and IgG1 levels, microtiter plates were coated for 2 h at room temperature with HDM (5 µg/mL) in 1 M carbonate buffer, pH 9.6. After three washings with PBS containing 0.05% (*v*/*v*) Tween-20 (PBS-T), plates were saturated for 1 h at 37 °C with a blocking buffer (PBS containing 0.5% (*w*/*v*) gelatin and 0.1% Tween-20). After three washings with PBS-T, plasma samples diluted in blocking buffer (1:20 for IgE and 1:200 for IgG1) were added into the wells of sensitized plates and left to incubate overnight at 37 °C. The plates were washed, and then anti-mouse IgE (Cliniscience, Nanterre, France clone 23G3-AP) or IgG1 (BD Biosciences, Le Pont-de-Claix, France, clone R85-3) in blocking buffer was added to the wells and incubated for 2 h at room temperature (RT). Plates were washed again. For IgE measurements, fluorescent substrate 4-methylumbelliferyl phosphate was added to the wells and incubated for 1.5 h at RT under light protection. The intensity of the reaction was measured using a fluorimeter (excitation 360 nm, emission 450 nm). For IgG1 measurements, HRP-anti-rat Ig conjugate (Abcam, ab6845-1) was added to the wells and left to incubate for 1 h at RT. The plates were washed and tetramethylbenzidine/H_2_O_2_ was added for 15–20 min. The reaction was stopped by adding 0.5 M H_2_SO_4_, and absorbance was measured at 450 nm. To analyse activity of P140 on Ig HDM-specific production in four studies, the results of each study are expressed in percentage of respective HDM groups.

For measuring total IgG1 and IgE levels, microtiter plates were coated overnight at 4 °C with anti-mouse IgG1 (BD Pharmingen, clone R85-3) or anti-mouse IgE (BD Pharmingen, clone R35-92) and antibodies were captured in 1M carbonate buffer, pH 9.6. After three washings with PBS-T, plates were saturated for 1 h at 37 °C with blocking buffer (PBS containing 1% SVF). After three washings with PBS-T, plasma samples diluted in blocking buffer (1:80 for IgE and 1:800 for IgG1) were added into the wells of coated plates and left to incubate 2 h at RT. The plates were washed, and then anti-mouse IgG1 (BD Pharmingen, clone A85-1) or IgE (BD Pharmingen, clone R35-72) in blocking buffer was added to the wells and incubated for 2 h at RT. The following steps and final revelation were performed as above.

### 2.6. Western Blotting

Measurement of autophagy marker expression in the spleen extracts and BAL cells was done by Western blotting as described [39,40]. The following antibodies were used: MAP1LC3B (MBL, M186-3), SQSTM1 (Abcam, ab109012), ATG5 (Cell Signaling Technology, 12994S), lysosome-associated membrane protein type 2A (LAMP2A; Abcam, ab125068), and HSPA8 (Abcam, ab51052). The secondary antibodies were HRP-conjugated goat anti-mouse or anti-rabbit IgG antibody (Jackson ImmunoResearch, Cambridge, UK, 115-035-008 and 111-035-008). Signal was detected using Clarity Western ECL Blotting Substrate (Biorad, 170-5061). The expression levels of autophagy markers were normalized by densitometry on ACBT (Santa Cruz Biotechnology, Heidelberg, Germany, sc-4778 HRP) level using ImageJ or Image Lab softwares. To evaluate the autophagic flux, the samples were incubated for 6 h with 100 nM bafilomycin A1 used as inhibitor of the autophagosome-lysosome fusion (this molecule inhibits acidification and protein degradation in lysosomes of cultured cells). The ratio of MAP1LC3B expression in cells with the inhibitor to the MAP1LC3B expression in cells without the inhibitor was measured. A ratio >1 corresponds to an increase of the autophagic flux and a ratio <1 suggests a diminution of the autophagic flux.

### 2.7. Statistical Analysis

Differences between groups were tested for statistical significance using one-way ANOVA followed by Tukey’s post-test. For two-group comparison studies, we used unpaired *t* test when *n* ≥ 8 per group or Mann–Whitney test when *n* < 8 per group. Data were considered significantly different when *p* ≤ 0.05.

## 3. Results

### 3.1. Effect of the Autophagy Regulator P140 in an Acute Model of OVA-Induced Eosinophilic Airway Inflammation

Based on our previous findings showing that P140 peptide significantly delays the inflammatory manifestations and mortality in several models of inflammatory autoimmune diseases (lupus, SS, CIDP), we first employed this peptide in an acute model of hypereosinophilic airway inflammation in mice, using OVA as the allergen [44]. A 15-day OVA-induced allergic-airway Th2 inflammation model in BALB/c mice was designed (Figure 1A). At day 0, mice were sensitized intraperitoneally with OVA adsorbed on alum. Sensitized mice of groups 2–4 were challenged via the i.n. route with OVA at days 5, 12, 13 and 14. Mice from group 1 received saline instead of OVA. Mice were treated either i.n. or i.v. with P140 (4 mg/kg body weight) given in an intuitive way at day 9, between two series of challenges. The choice for using 4 mg/kg was dictated by the dose of P140 used in our previous studies in mice [32,34,36,38] that showed beneficial effects of P140 used at this dosing. At day 15 (24 h after the last challenge), BALs were performed as described [46]. As expected, in OVA-challenged mice, the total number of cells recovered in BALF increased significantly (Figure 1B). This effect was related to a significant increased influx of eosinophils, neutrophils, and lymphocytes (*p* < 0.001). Administered locally by the i.n. route (group 3), P140 showed no detectable effect on inflammatory cell accumulation in BALs. In contrast, when given i.v. (group 4), P140 significantly decreased the accumulation of eosinophils (−50%; *p* < 0.001) and lymphocytes (−57%; *p* < 0.01) as well as neutrophil influx (−38%) although non-significantly (Figure 1B). These results show that a single i.v. dose of P140 strongly reduces airway eosinophilia and lymphocytosis. Comparing results from the 2 modes of administration of P140 (i.v. vs. i.n.) further suggests that P140 acts through a systemic (i.v.), rather than a local (i.n.), pathway in this model.

### 3.2. Effect of the Autophagy Regulator P140 in a Chronic Model of HDM-Induced Airway Inflammation

OVA is widely used to induce both acute and chronic experimental allergic asthma in mice. However, this model that also associates aluminum adjuvants is drastically different from the natural mode of sensitization in humans. We thus performed our next experiments using a chronic model of airway allergy that was induced by HDM, a clinically relevant allergen. Consecutive experiments were analyzed, of whose mice were sensitized and challenged with HDM extract given via the i.n. route; the other mice were controls that received no HDM but saline alone (Figure 2A). Control and HDM-sensitized mice received the P140 peptide, or the scrambled P140 peptide (ScP140) used as control, or the peptide vehicle alone, all given once by the i.v. route at day 22, just after the last challenge and relatively far (9 days) from the examination of tissues and BALs (Figure 2A).

We examined airway hyperresponsiveness (AHR) in these mice using the forced oscillation technique (FlexiVent) (Figure 2B). Nebulization of PBS caused no change in baseline airway resistance and elastance in saline or HDM-challenged mice treated with P140 or saline, whereas nebulization of MCh (50 mg/mL) increased airway reactivity (resistance and elastance) in all groups. HDM-sensitized mice as expected exhibited increased resistance and elastance, showing the presence of AHR, that was significantly regulated by treatment with P140, decreasing resistance by 77% (*p* < 0.01) and elastance by 64% (*p* < 0.05) (Figure 2B).

### 3.3. Effect of P140 Treatment on the Immune Cell Accumulation in the BALF Collected from HDM-Sensitized Mice

**Figure 3:**
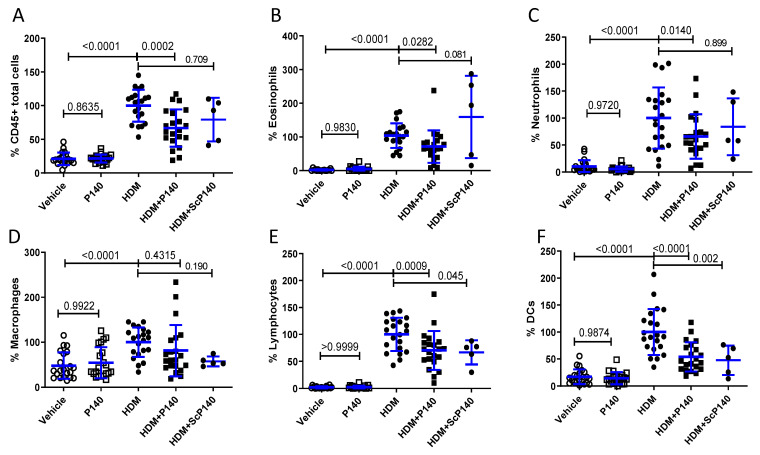
Effect of the P140 peptide on the cell content of BALFs collected from HDM-sensitized and challenged mice. 9-week-old female BALB/c mice were sensitized and challenged intranasally as indicated in Figure 2A. They receive vehicle, P140 or ScP140 intravenously at day 22, and BALFs were collected at day 31. The cell content of BALFs was studied by flow cytometry. (**A**–**F**) show the percentage of total CD45^+^ immune cells, eosinophils, neutrophils, macrophages, lymphocytes and DCs, respectively, expressed with regard to the average number of cells measured in the BALs of HDM-induced mice taken as 100%. The data are presented as the mean ± SEM (*n* = 5 or 21 mice per group from four independent experiments).

To determine to what extent this remarkable effect of P140 was linked to cell-driven inflammation in the lungs, we studied the cellularity of BALF collected from mice that received saline or HDM and were treated or not by P140 (Figure 3). Several independent experiments were performed to confirm the robustness of the P140 activity. As expected, the overall percentage of total CD45^+^ leukocytes was significantly raised in the BALF from HDM-sensitized and challenged mice (Figure 3A). This increased cell number was related to the influx of numerous cell subtypes in the lungs, especially eosinophils, neutrophils, macrophages, total lymphocytes and dendritic cells (DCs) (Figure 3B–F). Taking into account the results obtained with the control peptide ScP140 that displayed moderate but statistically significant effects on the percentage of BALF lymphocytes and DCs, the effect of P140 could be highlighted on eosinophils and neutrophils. The percentage of eosinophils and neutrophils was decreased upon P140 treatment by 35% (*p* = 0.0282) and 36% (*p* = 0.0140), respectively, with no concomitant effect of the ScP140 peptide (Figure 3B,C).

Probably because lung homogenates represent a complex mixture of a variety of cells and fluids, certain cell markers and soluble components remain difficult to detect. Thus, the effect of P140 on neutrophils present in the total fraction obtained by enzymatic digestion of lung tissue could not be visualized (not shown). Likewise, when a panel of nine cytokines (IL-1β, IL-4, IL-5, IL-6, IL-10, IL-13, TNF-α, CCL11 and CCL24) were tested by ELISA in total lung homogenates from HDM-sensitized and non-sensitized control mice sampled at day 31 (at sacrifice, i.e., 10 days after the last allergen challenge), no change could be visualized (Figure S1). Due to the lack of fluid needed to run the tests by ELISA, the levels of cytokines could not be evaluated in BALs. No conclusion on possible effects of P140 on cytokine/chemokine regulation could therefore be raised.

### 3.4. Effect of P140 on the Levels of Circulating Anti-HDM Antibodies

**Figure 4:**
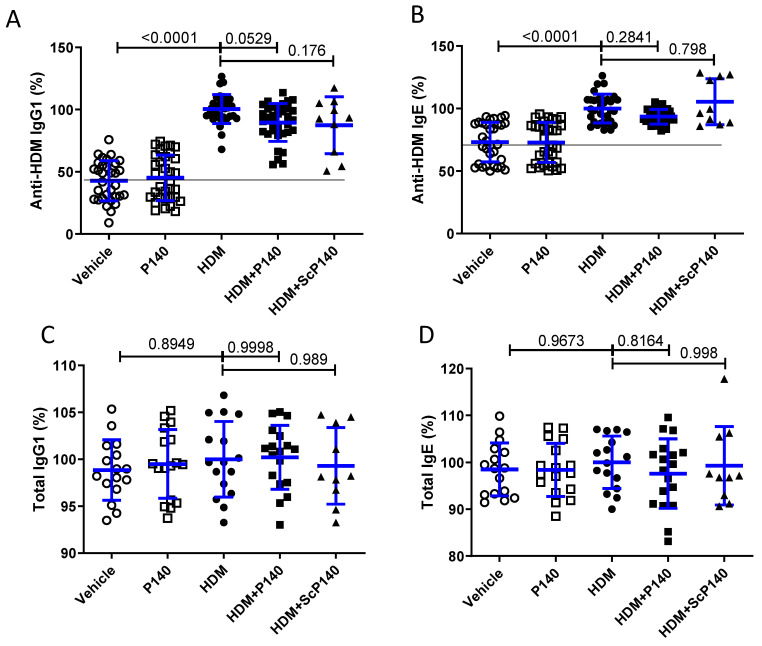
Effect of the P140 peptide on the levels of circulating anti-HDM IgG and IgE antibodies. The serum levels of specific IgG1 and IgE antibodies to HDM (**A**,**B**) and of IgG1 and IgE (**C**,**D**) were measured by ELISA. The level of Ig is expressed in percentage of respective HDM groups. The horizontal line indicates the background of the respective assays. The data are presented as the mean ± SEM (*n* = 32 mice per group from four independent experiments).

Our previous data in mice and humans [35,37,48] have shown that P140 does not exert any immunosuppressive action but rather behaves as an immunomodulator. To determine whether P140 affects IgG/IgE production in our chronic mouse model, we measured the circulating levels of specific anti-HDM IgG1 and IgE antibodies in the serum of mice treated by P140 or ScP140 or solvent (single injection at day 22, ELISA tests on plasma sampled at day 31). As expected, the serum levels of anti-HDM IgG1 and IgE antibodies were raised in mice that received HDM treated with solvent. The levels remained unaffected by P140 or ScP140 (Figure 4A,B). The serum levels of total IgG1 and IgE remained unmodified in all groups, treated or not with HDM and treated or not with P140/ScP140 (Figure 4C,D).

### 3.5. Effect of P140 on the Expression Profile of Autophagy Markers in the Spleen and BAL Cells of Model Mice with HDM-Induced Allergic Asthma

**Figure 5:**
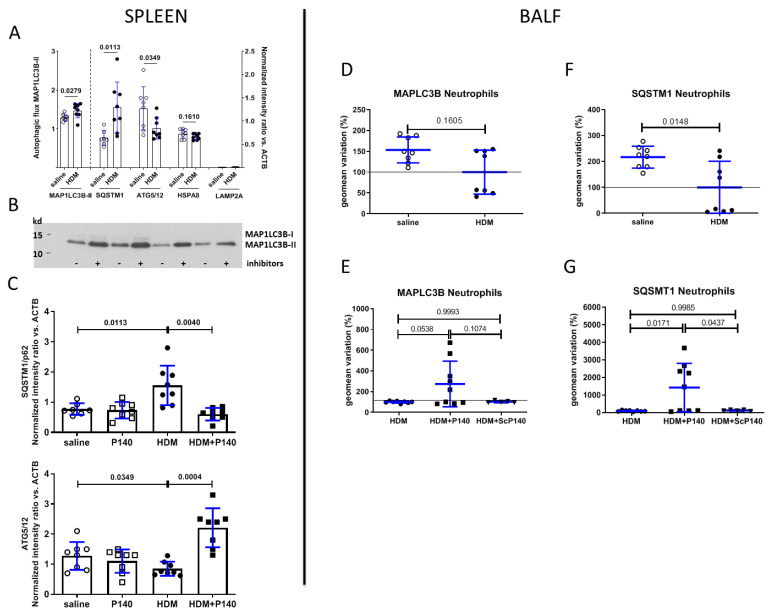
Measurement of autophagy markers in total splenocytes and BAL neutrophils collected from mice from HDM-sensitized and challenged mice. Nine-week-old female BALB/c mice were sensitized and challenged intranasally as indicated in Figure 2A. They receive vehicle, P140 or ScP140 intravenously at day 22; BALFs and spleens were collected at day 31. (**A**,**B**) Western immunoblotting and semi-quantification of five autophagy markers present in the spleen extracts from mice that received saline only or that were sensitized and challenged by HDM, and were treated or not with P140 (*n* = 8 in each group). The data are presented as the mean ± SEM. In (**A**), the autophagic flux (**left** part) corresponds to the ratio of MAP1LC3-II protein expression found in cells incubated in the presence or absence of bafilomycin A1. In (**A**) (**right** part) and (**C**), the expression of the other proteins, as measured by densitometry of Western blots, was normalized with regard to the β-actin/ACTB level. (**D**–**G**) Flow cytometry semi-quantification of autophagy markers present in the neutrophil population present in the BALF from mice that received saline only or that were sensitized and challenged by HDM with or without treatment with P140. Two markers were tested, namely MAP1LC3B (**D**,**E**) and SQSTM1 (**F**,**G**). The results generated with other cell types are shown in the Figure S2. The data are represented as the mean variation ± SEM of the geomean (in%). The horizontal lines indicate the 100% level. Saline, *n* = 8; HDM, *n* = 8; HDM + P140, *n* = 9; HDM + ScP140, *n* = 5. Pooled data from 2 independent experiments.

Our past experiments in the lupus context, both in vitro and in vivo, have shown that P140 enters MRL/lpr spleen B cells via a clathrin-dependent pathway and accumulates into lysosomes [33]. Furthermore, the levels of HSPA8 chaperone protein and LAMP2A, which are both overexpressed in MRL/lpr B cells, were found to be corrected after P140 treatment of MRL/lpr lupus-prone mice [32,34]. Since some data argue that autophagy processes are activated in the lungs of subjects with asthma [28], we have sought first to identify and quantify the extent of autophagy defaults in mice with HDM-induced allergic airway features (Figure 5A,B,D,F) and second, to examine the capacity of P140 peptide to restore the basal level of autophagy activity by treating HDM-sensitized and -challenged mice (Figure 5C,E,G). These studies were performed both in the spleen and in BALFs. The gating strategy for immune cell subsets is illustrated in Figure S3.

Immunoblotting experiments with spleen extracts showed that the expression of HSPA8 and LAMP2A is not affected by HDM sensitization with regard to the basal level of expression of these markers in BALB/c mice that received saline only instead of HDM (Figure 5A). However, the autophagic flux as measured by the expression of MAP1LC3B-II in the presence and absence of protease inhibitors (*p* = 0.0279) and expression of SQSTM1 (*p* = 0.0349) and ATG5/12 complex (*p* = 0.0113) proteins was significantly altered, raised in the case of autophagic flux and SQSTM1 and decreased in the case of ATG5/12 (Figure 5A,B). Immunoblotting experiments performed with splenic extracts of HDM-sensitized mice further revealed that the abnormal expression of both autophagy markers recovered their basal expression level after a single i.v. injection of P140 given at day 22 (*p* = 0.0040 and 0.0004, respectively; Figure 5C).

Testing autophagy markers in the inflammatory cells recovered in the BALF of HDM-sensitized mice by Western blotting was not possible due to too low a number of cells in regard to the sensitivity of the immunoblotting test. We thus set up new cellular assays to evaluate the expression of autophagy markers of BAL cells by flow cytometry. Beyond its sensitivity, this technique allowed us to examine the expression of autophagy markers in various BALF cell subtypes. Two markers only could be followed, namely MAP1LC3B and SQSTM1 (Figure 5D–G and Figure S2). Compared to control mice that received no HDM but saline only, the expression of MAP1LC3B remained unchanged upon HDM sensitization and challenge in any cells of the BALF (shown as example in neutrophils, Figure 5D) with no statistically significant effect of P140 and ScP140 (shown in neutrophils where a non-significant trend was observed; Figure 5E). However, the expression of SQSTM1 was decreased by half on average upon HDM stimulation in neutrophils (Figure 5F; *p* = 0.0148) of the BALs. The expression level of SQSTM1 was also reduced in lymphocytes, monocytes and DCs of the BALs but increased in eosinophils (Figure S2A). These expression changes of SQSTM1 were not rescued by P140 treatment in lymphocytes, monocytes, DCs and eosinophils of the BALs (Figure S2B). However, upon P140 treatment, the reduced level of expression of SQSTM1 (that seems to affect some mice but not all; Figure 5F) was significantly corrected and particularly raised in BALF neutrophils of HDM-sensitized mice (Figure 5G; *p* = 0.0171), while the control peptide ScP140 showed no effect. Further phenotyping analyses would be required to determine the maturation state of the different neutrophil subtypes that are SQSTM1^high^ and to pin down their kinetics of appearance and lifespan.

## 4. Discussion

In the present study, we provide novel insights into the potential of autophagy modulators in chronic inflammatory diseases as asthma. Our results show that a single administration of P140 given i.v. in HDM-sensitized and -challenged mice was sufficient to temper inflammation, and allow them to recover their lung capacities in a few days. Respiratory mechanic parameters (airway resistance and tissue elastance) confirmed disease-specific phenotypes of HDM-sensitized and challenged P140-treated BALB/c mice following MCh challenge that are all regulated by P140 treatment.

This work was performed using two well-recognized models of allergen-induced airway inflammation in mice and demonstrated the anti-inflammatory activity of the therapeutic peptide P140 in this setting. First, we evaluated the P140 anti-inflammatory activity in an acute model of OVA-induced eosinophilic airway inflammation and found that intravenous administration of P140 significantly decreased eosinophils and lymphocytes accumulation in BALFs. Based on these findings, we then evaluated P140 activity using the HDM-induced allergic airway inflammation model. Evaluating experimental therapeutics using this model was pivotal in our study, as it could lead to new options for asthma patients whose symptoms are not well managed with current treatment options [49]. HDM is an immunologically complex allergen, which can stimulate both the innate and adaptive immune responses. Contrarily to the OVA-induced model, the HDM-induced allergic model does not require aluminum hydroxide as an adjuvant and more closely resembles the heterogeneous nature of human allergic asthma. It is effectively well documented that alum, a conventional adjuvant that is approved for human vaccination for decades, can trigger the release of NETs by neutrophils [50,51], a feature that could obscure or even alter the effect of P140 peptide. The chronic HDM model is not only a more natural model that is closer to human pathophysiology, but is also free of this potential bias. In our experiments, it was characterized by an accumulation of inflammatory cells in BALFs and a sustainable AHR ten days after the last allergen challenge. Alteration of autophagy could be identified in cell subsets known to display key roles in asthma. The beneficial activity of the P140 peptide was confirmed in the HDM model at the clinical level. We especially described a significant benefit of P140 on AHR and demonstrated some correcting effects of P140 on the expression of autophagy markers.

Our findings highlight that defects exist in the autophagy pathway of mice that develop chronic allergic asthma induced by HDM. These defaults, which mostly imply the autophagy marker SQSTM1, appear both in the cells that invade the lungs of certain affected mice and in the spleen, a peripheral lymphoid organ, which maintains mature naive lymphocytes and initiates an adaptive immune response. Interestingly, expression of BECN1 and ATG5 has been found to be increased, together with reduced expression of SQSTM1, in the large airway smooth muscle of patients with asthma compared with healthy individuals without asthma [28]. Our results further indicate that P140 regulates the abnormal expression of SQSTM1 that for yet unknown reasons that could be to do with transient, compensatory mechanisms, is downregulated in neutrophils present in the BALF and is overexpressed in the spleen. The effects were detectable in BAL cells a few days only after a single administration of P140 given to mice developing chronic inflammatory disease of the airways after a repetitive cycle of sensitization and challenges. As visualized in the spleen of asthma-like animals, the effect of P140 seems to occur also at the systemic level, where it also focuses its principal activity towards neutrophils. Overall, these findings strongly reinforce the potential of autophagy regulators, such as P140, for treating selected patients with asthma.

The extent of autophagy dysregulation is still poorly understood in asthma. Here we found opposite autophagy anomalies in BAL cells and splenocytes of sick mice. We already pointed out the fact that autophagy activity can be increased in certain organs, tissues or cells, while it can be abnormally decreased in others of a same individual. It was the case, for example, when we studied the autophagy activity extent in the spleen and salivary glands of model mice with SS [36]. In this model, likewise, P140 corrected the two opposite defaults. Additional experiments will be needed in asthma to identify the primary site of peptide drug intervention. It seems here that since in the OVA model P140 exerted no effect when delivered via the i.n. route, while it had potent effects when given i.v., its action might be rather systemic and may involve organs other than the proper pathophysiological site in the lung.

SQSTM1 is a multifunctional, stress-induced, signaling molecule, involved in a variety of cellular pathways, including signaling pathways such as nuclear factor of *kappa* light polypeptide gene enhancer in B-cells (NF-κB) activation, nerve growth factor signaling, and caspase activation. It is also one of the best-known autophagic substrates and is widely used as a marker of autophagic flux. SQSTM1 interacts with a central component of the autophagy machinery, MAP1LC3 [52], and transports altered proteins to degradation by autophagy. It is best characterized to mediate autophagic clearance of polyubiquitinated cargos such as aggregated proteins. Its intracellular accumulation can have several meanings, and in particular indicates an inefficient (or blocked) autophagy. Conversely, its lower expression can be indicative of an intense autophagy activity. These features, however, remain largely cell- and condition-specific, and interpreting the reasons of SQSTM1 accumulation or defects may prove difficult. Intracellular levels of SQSTM1 are controlled through a turnover by autophagy, but also at the transcriptional level upon insults such as oxidative, proteotoxic or endoplasmic reticulum stress, making the understanding of changes in SQSTM1 protein amounts even more complex.

Our observations that the number of neutrophils, which is raised in the BALFs collected after HDM activation, is recovered upon P140 treatment and that neutrophilic autophagy activity and is diminished in sick animals and significantly restored—even exacerbated in some mice—by P140 treatment are interesting to address. The fact that neutrophils can act as antigen-presenting cells (APCs) and bring MHC-II molecules at their surface has long been underestimated [53]. As in lupus in which B cells act as APCs, it is possible that here also P140 affects antigen-presenting capacities of neutrophils by destabilizing its receptor, the chaperone HSPA8, leading to a lowering of overexpression of antigens by MHC-II molecules to T cells, and a normalization of functions of cells of the innate and adaptive immune system. In lupus, it seems that this intervention pathway operates via an effect on CMA that is over-activated in APCs [33]. It remains to be determined more finely whether CMA or other forms of autophagy are abnormally activated in neutrophils of the BALs and other tissues/organs in allergic asthma. In this regard, it is important to remember that autophagy is not only involved in neutrophil differentiation, but also plays crucial effector functions of neutrophils, such as granule formation, degranulation, NET release, cytokine production, bactericidal activity and control of inflammation [54]. Autophagy is also decisive in the biology of eosinophils with a central effect on degranulation, for example [31]. If some of these effects as the release of NETs, toxic proteins, and certain proteases could be damaging for the lung, especially if they occur in a non-controlled context, apoptotic death has multiple pro-resolution actions. Apoptotic inflammatory cells become unresponsive to agonists, stop the production of inflammatory mediators and can sequester cytokines with beneficial effects to regulate inflammation. Moreover, phagocytosis of neutrophils by macrophages may promote macrophage polarization from a pro-inflammatory (M1) to a pro-resolution (M2) [55].

Real-time bioimaging experiments in living MRL/lpr mice have shown previously that, rapidly upon i.v. administration, AF-labelled P140 accumulates in the lungs and spleen of pre-lupus and lupus mice [32]. HSPA8 to which P140 readily binds [56] is also highly expressed in these two organs (in human tissues, 4.7 times more in the spleen as compared to lungs [57]). An interesting question that remains to be explored is to determine if, as found at the surface of splenocytes and lymph node cells of lupus mice [32], HSPA8 is also overexpressed in specific cells, especially neutrophils, in the inflamed lungs and spleen of HDM-treated mice, and whether P140 also rescues mice from this higher expression. In the present context, the fact that HSPA8 could be increased at the surface of certain inflammatory cell subsets after HDM sensitization would be beneficial for therapy as, like a first favorable flag, it could target P140 to specific inflamed tissues and avoid P140 entering in any (non-affected) cells.

## 5. Conclusions

Today, asthma represents a serious issue of public health. This remains because of the growing number of patients that are affected (hundreds of millions of people are afflicted worldwide [56]) and beyond this, by the numerous asthma “endotypes-phenotypes” that co-exist [58,59]. The complexity of asthma in terms of origin (triggering and challenging allergen), type of lung inflammation (e.g., neutrophilic or eosinophilic asthma), severity, natural history, comorbidities, and treatment response, which varies with time, is extremely large. There is an urgent need for defining novel ways of intervention, in general long-term therapies, to anchor expectations of patients—often in early childhood—and medical professionals. Personalized medicine has been proposed to respond more precisely to specific pathophysiological mechanisms that are underlying each condition [59]. Here, we show that P140 exerts a beneficial effect in two unrelated mouse models of allergic airway inflammation, induced by acute OVA and chronic HDM. It remains to be explored whether non-Th2 asthma conditions can also benefit from a potential peptide drug such as P140, as it does not target a specific allergen-induced mechanism but a metabolic pathway, the exacerbation of which results into an inflammatory response, likely due here to allergen exposure, in predisposed patients [9]. Other indications involving autophagy, such as acute respiratory distress syndrome or other viral or non-viral respiratory failures, might also benefit from this therapeutic peptide-based strategy [60,61].

## Data Availability

The raw data supporting the conclusions of this article will be made available by the authors, without undue reservation.

## Supplementary Materials

The following results are available online at https://www.mdpi.com/article/10.3390/cells10092468/s1, Figure S1: ELISA measurement of cytokines/chemokines in lung extracts of mice of the four study groups, Figure S2: Measurement of the autophagy marker SQSTM1 in the cells of BALFs collected from mice from HDM-sensitized and challenged mice.
