# Supplementary material for: An Autophagy Modulator Peptide Prevents Lung Function Decrease and Corrects Established Inflammation in Murine Models of Airway Allergy
Source: Cells. 2021 Sep 18;10(9):2468. doi: 10.3390/cells10092468 (PMC8472429; doi:10.3390/cells10092468)
Supplement: Supplementary file 1 [file cells-10-02468-s001.zip › cells-1366519-supplementary.pdf]

Supplementary Materials

An Autophagy Modulator Peptide Prevents Lung Function Decrease and Corrects Established Inflammation in Murine Models of Airway Allergy

François Daubeuf [1,2], Nicolas Schall [3], Nathalie Petit-Demoulière [1,3], Nelly Frossard [1] and Sylviane Muller [3,4,5,*]

[1] CNRS-Strasbourg University Laboratoire d'Innovation Thérapeutique/Strasbourg Drug Discovery and Development Institute (IMS), Faculté de Pharmacie, 67400 Illkirch, France; francois.daubeuf@unistra.fr (F.D.); nathalie.petit-demouliere@unistra.fr (N.P.-D.); nelly.frossard@unistra.fr (N.F.)

[2] CNRS UMS3286, Plate-forme de chimie biologique intégrative de Strasbourg/Strasbourg Drug Discovery and Development Institute (IMS), 67400 Illkirch, France

[3] CNRS-Strasbourg University Unit Biotechnology and Cell signaling/Strasbourg Drug Discovery and Development Institute (IMS), Strasbourg, France; Ecole Supérieure de Biotechnologie de Strasbourg, 67400 Illkirch, France; n.schall@unistra.fr

[4] Fédération Hospitalo-Universitaire OMICARE, Fédération de Médecine Translationnelle de Strasbourg, Strasbourg University, 67000 Strasbourg, France

[5] University of Strasbourg Institute for Advanced Study, 67000 Strasbourg, France

* Correspondence: sylviane.muller@unistra.fr

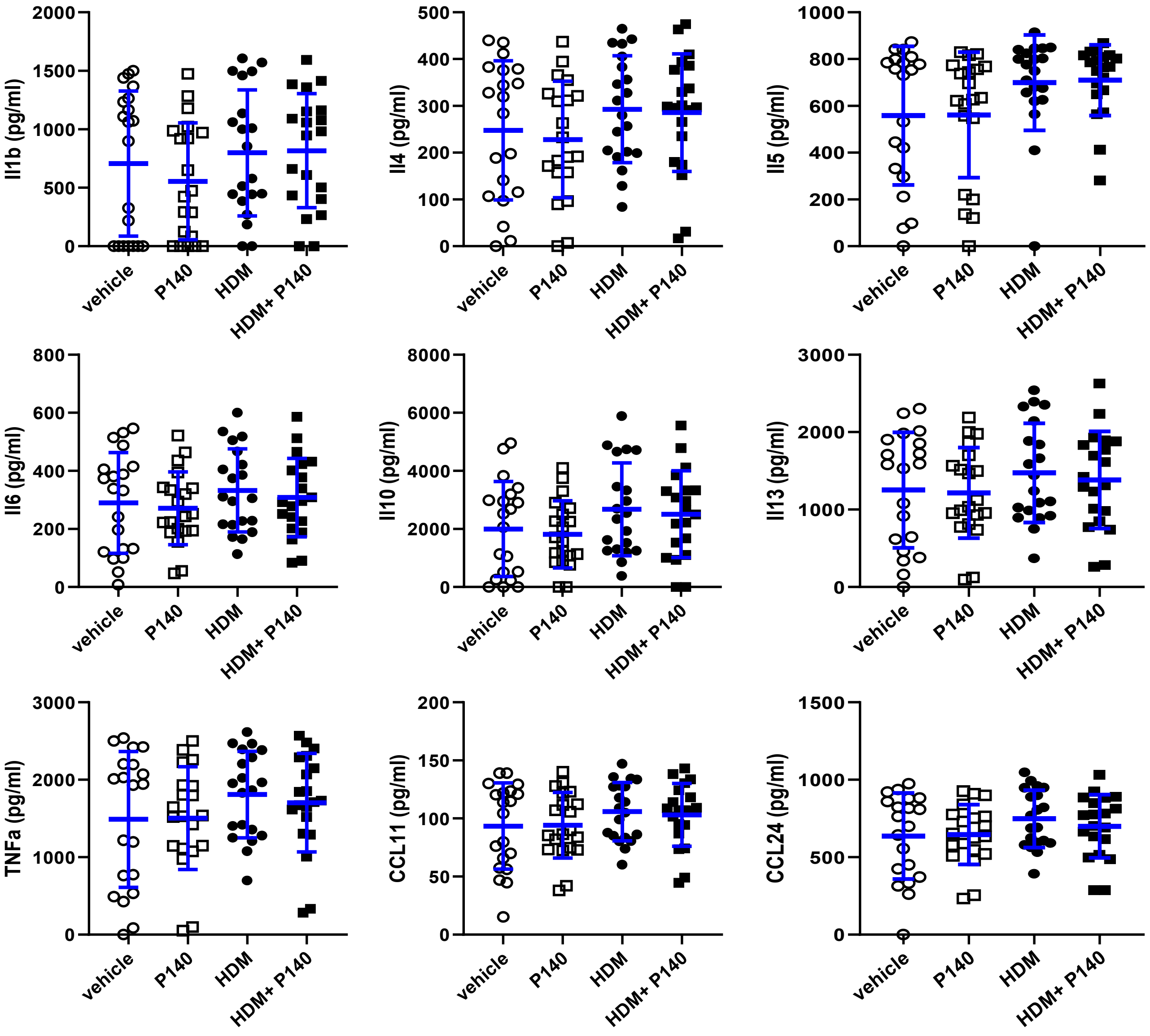

Supplementary Figure S1. ELISA measurement of cytokines/chemokines in lung extracts of mice of the four study groups (n = 20). The data are presented as means ± SEM. Differences between groups were tested for statistical significance using one-way ANOVA followed by Tukey's post-test. Data were considered significantly different when $p \leq 0.05$.

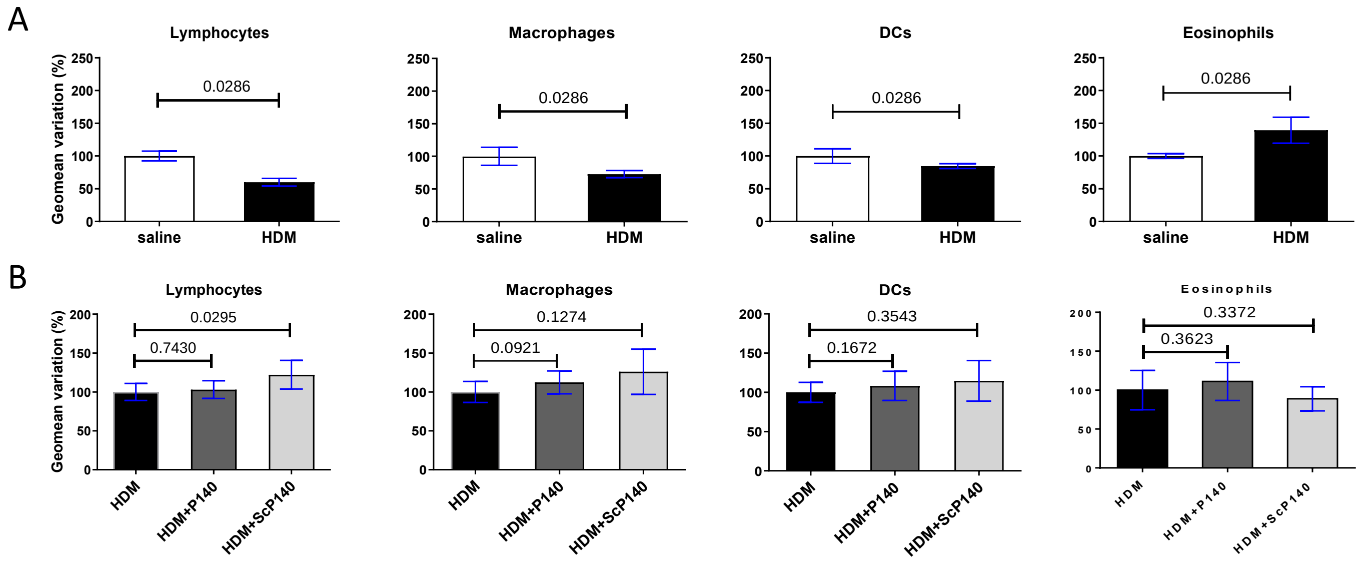

Supplementary Figure S2. Measurement of the autophagy marker SQSTM1 in the cells of BALFs collected from mice from HDM-sensitized and challenged mice. 9-week-old female BALB/c mice were sensitized and challenged intranasally with HDM as indicated in Figure 2A. They receive vehicle or P140 intravenously at day 22; BALFs were collected at day 31. SQSTM1 expression was measured by flow cytometry in different cell subsets. (**A**) Effect of HDM versus saline. (**B**) Effect of P140 (and ScP140 control peptide) versus vehicle in HDM-induced mice. The data are represented as the mean variation ± SEM of the geomean (in%). HDM, n = 8 ; HDM + P140, n = 9 ; HDM + ScP140, n = 5 (pooled data from 2 independent experiments).

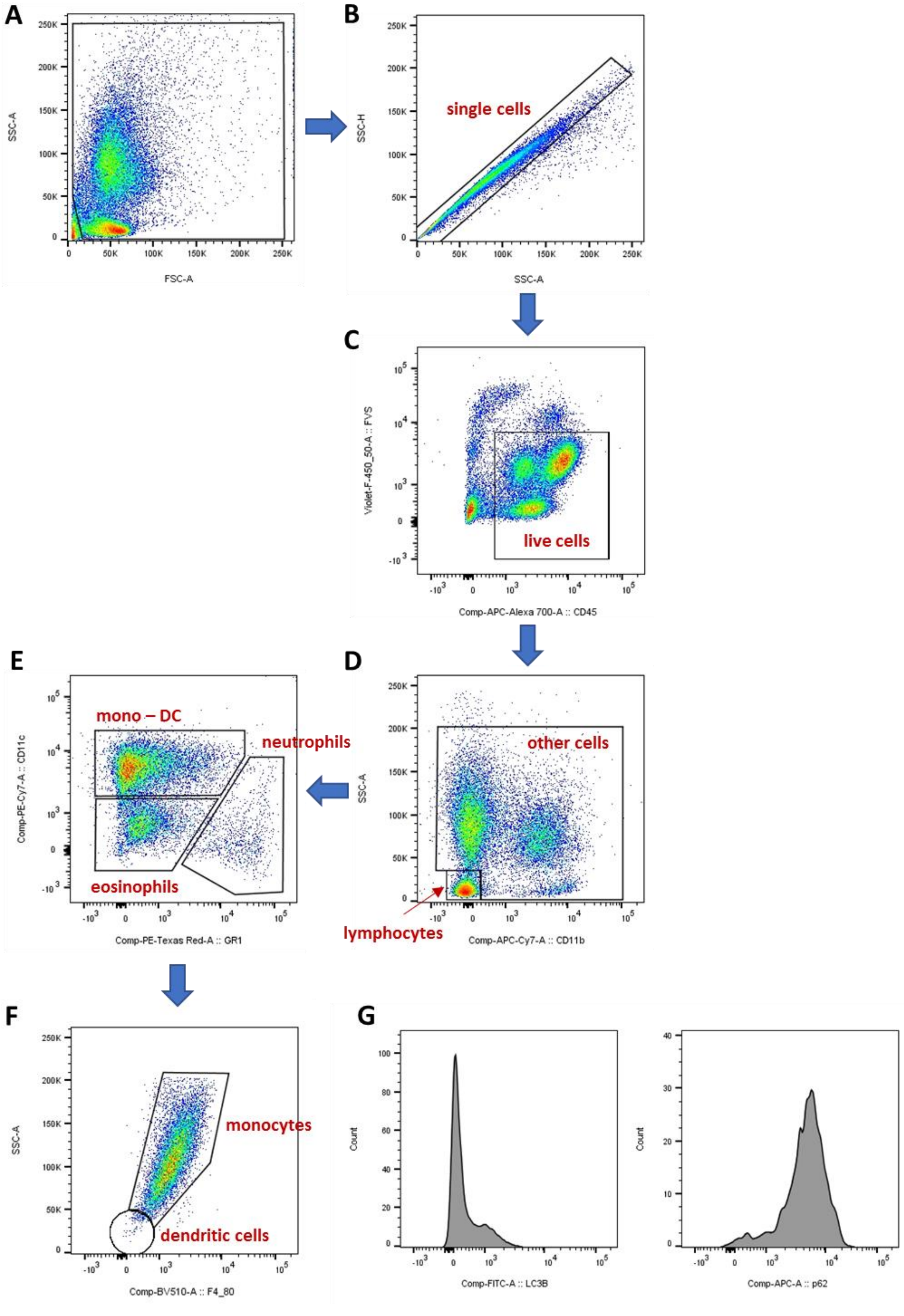

Supplementary Figure S3. Gating strategy for immune cell subsets in BALF and spleen. Cells were gated in SSC-A and FSC-A dot blot to eliminate debris (**A**). Then cells were gated in a SSC-H and SSC-A dot blot to eliminate doublets (**B**). CD45+ cells were chosen to analyze live leucocytes (**C**). CD11b+ cells were chosen to analyze mononuclear cells (**D**). Gr-1 and CD11c+ cells were chosen to analyze monocytes and dendritic cells, eosinophils and neutrophils (**E**). F4/80 signal was used to distinguish dendritic cells from monocytes in BAL cell population (**F**). LC3B-II and SQSTM1/p62 expression was studied in these cell subsets (**G**). Abbreviations: FSC: Forward scatter; FSC-H: Forward scatter height; FSC-A: Forward scatter area; SSC: Side scatter.
